# Omics approaches to unveil biotic stress responses in olive: current knowledge and the future

**DOI:** 10.1093/jxb/erag337

**Published:** 2026-07-08

**Authors:** Aparna S Balan, Antonio Giovino, Tiziano Caruso, Annalisa Marchese

**Affiliations:** Department of Agricultural, Food and Forest Sciences (SAAF), University of Palermo, Palermo, Italy; Council for Agricultural Research and Economics (CREA)—Research Centre for Plant Protection and Certification (CREA-DC), Palermo, Italy; Department of Agricultural, Food and Forest Sciences (SAAF), University of Palermo, Palermo, Italy; Department of Agricultural, Food and Forest Sciences (SAAF), University of Palermo, Palermo, Italy; University of Ghent, Belgium

**Keywords:** Epigenomics, metabolomics, metatranscriptomics, pathways, RNA-seq, transcriptomics

## Abstract

Olive (*Olea europaea*) is a perennial crop of major economic, cultural, and ecological importance in the Mediterranean basin and worldwide, yet its productivity and sustainability are increasingly constrained by diverse pathogens, including fungi, bacteria, and viruses. The primary objective of this review is to critically evaluate how recent omics advances have improved our understanding of olive immune regulation under biotic stress, and to clarify why an integrative systems-level multi-omics perspective is essential for developing durable disease management strategies. By synthesizing genomic, transcriptomic, and emerging proteomic, microbiomic, and metabolomic studies, we show that olive defense relies on tightly coordinated transcriptional reprogramming, hormone crosstalk, metabolic remodeling, and structural barriers, within a multilayered immune framework, supported by expanding genomic resources and resistance-associated loci in cultivated and wild germplasm. However, the review also identifies major limitations that currently hinder progress, including a predominant transcript-centric focus, limited proteomic, microbiomic, and metabolomic depth, insufficient attention to post-translational and epigenomic regulation, underdeveloped pan-genomic frameworks, and scarce functional validation using genome-editing approaches. We conclude that integrating multilayered omics with functional genomics and pan-genomics is crucial for translating molecular insights into resilient cultivars and sustainable disease management, and we outline key priority directions for future research and breeding applications.

## Teaser

Omics-based approaches reveal regulatory networks underlying olive immune responses, identify critical knowledge gaps, and propose a systems-level roadmap for functional validation and development of resistant cultivars.

## Introduction

Olive (*Olea europaea* L.) is one of the oldest cultivated tree crops and is now grown worldwide due to its adaptability to diverse agro-climatic conditions (Julca *et al*., 2020). Beyond its economic value for olive oil and table production, the olive plays a crucial ecological role in semi-arid environments, contributing to soil conservation and sustainable agroecosystems. Moreover, the persistence of centennial and even millennial olive trees highlights the remarkable longevity, genetic resilience, and adaptive capacity of this species under prolonged environmental and biotic pressures (Marchese *et al*., 2023a).

Despite this environmental resilience, olive cultivation is increasingly threatened by fungal and bacterial pathogens that compromise productivity, oil quality, and orchard longevity. Major diseases include peacock spot caused by *Spilocaea oleaginea*, Verticillium wilt caused by *Verticillium dahliae*, olive knot induced by *Pseudomonas savastanoi*, anthracnose associated with *Colletotrichum* spp., and Olive Quick Decline Syndrome linked to *Xylella fastidiosa* (*Xf*) subsp. *pauca* (Saponari *et al*., 2013; Luvisi *et al*., 2017b; Keykhasaber *et al*., 2018; Martinelli *et al*., 2019; Talhinhas *et al*., 2018; Licciardello *et al*., 2023; Marchese *et al*., 2023b). These pathogens differ in epidemiology and infection strategy, ranging from foliar necrotrophs to vascular colonizers, yet collectively threaten the sustainability of olive production under changing climatic conditions. Consequently, omics-based approaches are increasingly used to unravel host–pathogen interactions, identify resistance mechanisms, and accelerate the development of disease-resilient cultivars.

Like other higher plants, olive relies on a multilayered immune system in which physical and chemical barriers (cuticle, cell wall, and antimicrobial secondary metabolites) form the first line of defense. In this context, resistance refers to the ability to restrict pathogen establishment and/or proliferation, whereas tolerance describes the capacity to maintain physiological and metabolic functions despite infection (Pagán and García-Arenal, 2018). Pathogen recognition activates pattern-triggered immunity (PTI) via pattern recognition receptors (PRRs), inducing rapid reactive oxygen species (ROS) production, signaling cascades, and defense gene expression; when effectors suppress PTI, effector-triggered immunity (ETI) can be activated through intracellular resistance proteins, leading to stronger localized responses (Castañeda-Ojeda *et al*., 2017; Moretti *et al*., 2021). These responses are regulated by hormonal networks involving salicylic acid (SA), jasmonic acid (JA), ethylene (ET), and abscisic acid (ABA), together with transcriptional and post-transcriptional regulators that fine-tune defense gene expression (Gómez-Lama Cabanás *et al*., 2015; Gharbi *et al*., 2017; Cardoni *et al*., 2022; Marchese *et al*., 2023b). Overall, olive resistance is predominantly quantitative and cultivar dependent, reflecting coordinated structural, metabolic, and regulatory adjustments rather than single major resistance genes.

Climate change further exacerbates these challenges by influencing pathogen distribution, host susceptibility, and disease severity (Montes-Osuna and Mercado-Blanco, 2020; Morelli *et al*., 2021; Poveda and Baptista, 2021). The perennial nature and long life span of olive trees complicate disease management, as infections may persist and spread within orchards over extended periods. Traditional control measures often show limited efficacy and raise environmental concerns, reinforcing the need for genetically based, durable resistance strategies (Basile *et al*., 2023; Lantero *et al*., 2023).

In recent years, advances in genomics, transcriptomics, and other omics technologies have generated valuable resources for dissecting olive–pathogen interactions. High-quality genome assemblies, resequencing datasets, and RNA sequencing (RNA-seq) studies have identified defense-associated pathways involving hormone signaling, phenylpropanoid metabolism, oxidative stress regulation, and vascular remodeling. Nevertheless, most studies remain largely descriptive and transcriptome-centered, with limited integration of proteomic, metabolomic, and phenotypic data into predictive frameworks.

Emerging research on olive highlights the growing use of integrated omics to better understand complex stress responses. Multi-omics approaches have been applied to salt stress in olive, showing how transcriptomic, proteomic, and metabolomic integration provides a more complete view of adaptation mechanisms (Claros *et al*., 2025). Similarly, omics studies in table olives have used data integration to characterize microbial and biochemical dynamics (Randazzo *et al*., 2017). However, integrative omics approaches in olive biotic stress research remain limited, highlighting a significant gap. This review addresses this by synthesizing current omics-based knowledge of olive defense responses to major pathogens, critically evaluating existing limitations, and outlining strategies to translate molecular insights into practical applications for resistance breeding and sustainable olive production.

## Overview of plant immune responses in olive

Unlike annual model plants, where single resistance genes often determine host–pathogen compatibility, olive defense is a multilayered and dynamic system. It integrates constitutive structural barriers, inducible immune signaling, and extensive metabolic reprogramming. Olive tissues exhibit strong baseline defenses, including lignin- and phenolic-rich cell walls and antimicrobial secondary metabolites such as oleuropein derivatives, which together create physical and biochemical barriers limiting pathogen penetration and colonization (Fig. 1A). These constitutive traits are particularly important against vascular pathogens such as *V. dahliae* and root-associated oomycetes, where resistant cultivars maintain tissue integrity and vascular functionality during infection (Luvisi *et al*., 2017a; Saponari *et al*., 2019; Mascuñano *et al*., 2025). High basal levels of phenolic metabolites from the phenylpropanoid pathway, including oleuropein, hydroxytyrosol derivatives, and flavonoids, further contribute to antimicrobial activity and redox homeostasis. Complementing these structural defenses, olive mounts inducible immune responses upon pathogen perception, conceptually framed as PTI and ETI. Contrary to many annual model species, olive relies heavily on constitutive defenses operating over long temporal scales, reflecting its perennial growth habit and prolonged exposure to diverse biotic challenges. Taken as a whole, the evidence supports the notion that olive defense relies predominantly on sustained PTI-associated signaling and metabolic adaptation, with comparatively limited experimental evidence supporting canonical ETI, highlighting the importance of integrated, multilayered defense strategies in this long-lived woody species (Fig. 1). This emerging view is consistent with the predominance of quantitative resistance observed across olive pathosystems, where disease outcomes appear to depend on the cumulative contribution of multiple defense mechanisms acting in concert.

**Fig. 1.**
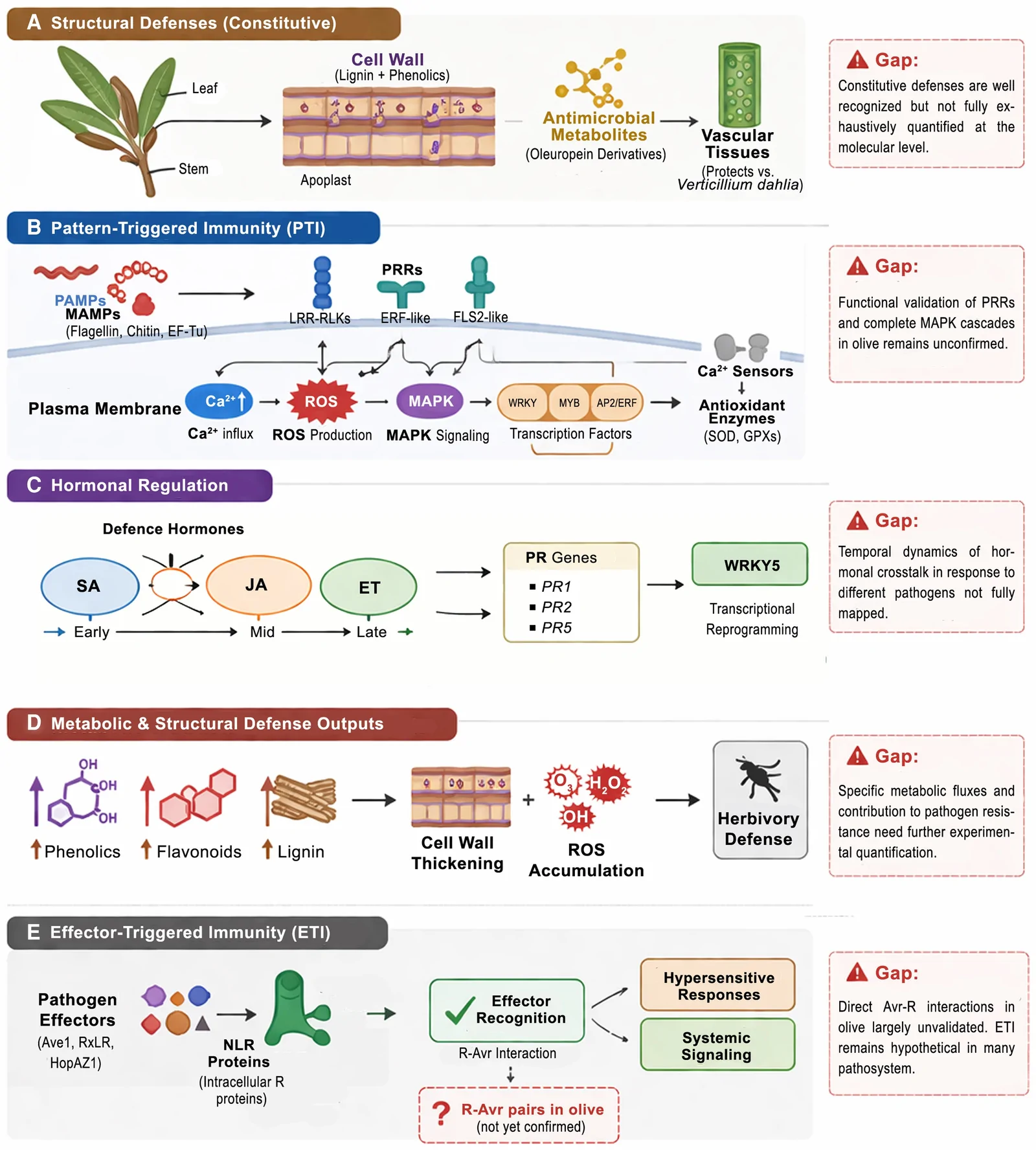
Multilayered defense strategies in olive (*O. europaea*) depicting structural, molecular, hormonal, and metabolic defenses against pathogens, including pattern-triggered immunity (PTI) and effector-triggered immunity (ETI), with highlighted knowledge gaps indicating areas where mechanisms remain incompletely characterized or experimentally unvalidated. (A) Structural defenses (constitutive): lignin- and phenolic-enriched cell walls, along with oleuropein derivatives in the apoplast, provide basal protection against pathogens such as *V. dahliae*. (B) PTI: plasma membrane receptors detect PAMPs, activating Ca^2+^ influx, ROS production, MAPK signaling, and transcription factors (WRKY, MYB, AP2/ERF). (C) Hormonal regulation: SA (salicylic acid), JA (jasmonic acid), and ET (ethylene) coordinate defense gene expression (*PR1*, *PR2*, *PR5*) and transcription factors (WRKY5) over time. (D) Metabolic and structural defense outputs: accumulation of phenolics, flavonoids, lignin, ROS (reactive oxygen species), and cell wall thickening enhances defense, including protection against herbivory. (E) ETI: NLR receptors recognize pathogen effectors (Ave1, RxLR, HopAZ1), triggering the hypersensitive response and systemic signaling, with some interactions remaining unconfirmed.

### Pattern recognition and early immune signaling

The first layer of inducible plant immunity is based on the recognition of pathogen-associated molecular patterns (PAMPs) by plasma membrane-localized PRRs, including receptor-like kinases (RLKs) and receptor-like proteins (RLPs), which activate PTI and restrict pathogen establishment (Fig. 1B; Movahedi *et al*., 2022); however, in olive, evidence for these components is currently derived mainly from transcriptomic analyses, and their functional roles in PTI have not yet been experimentally validated. Consequently, current models of pathogen perception in olive rely primarily on comparative genomics and expression profiling, highlighting a substantial gap between candidate receptor identification and functional validation. Several RLKs and RLPs have been identified in olive as putative components of PTI, although their functions are largely inferred from homology and expression data. Leucine-rich repeat (LRR) receptor-like serine/threonine kinases homologous to canonical PRRs such as FLAGELLIN SENSING 2 (FLS2), which recognizes flg22, and ELONGATION FACTOR TU RECEPTOR (EFR), which recognizes EF-Tu, have been detected in olive genomic and transcriptomic datasets, with an *FLS2*-like gene showing sequence similarity and pathogen-induced expression, suggesting conserved PAMP perception mechanisms (Estudillo *et al*., 2025). In addition, multiple LRR-RLKs, G-type lectin S-RLKs, and LysM- and malectin-like kinases are differentially expressed or accumulated during interactions with pathogens such as *S. oleaginea* and *V. dahliae* (Giampetruzzi *et al*., 2016; Marchese *et al*., 2023b). RLPs are also present in the olive genome, but none of these RLK/RLP candidates has yet been functionally validated as a PRR through ligand binding or reverse genetics, leaving PRR-mediated perception in olive largely based on indirect evidence (La Notte *et al*., 2024; Fig. 1B).

As a key second messenger in downstream defense responses, cytosolic Ca^2+^ influx is rapidly triggered by PTI activation, representing one of the earliest signaling events following PRR activation. In olive roots infected with *V. dahliae*, transcriptomic analyses show early induction of Ca^2+^-related genes, including those encoding calmodulins, calcium-dependent protein kinases (CDPKs), and Ca^2+^ transporters such as Ca^2+^ ATPases and Ca^2+^ exchangers, indicating a rapid and coordinated calcium response. This Ca^2+^ burst is decoded by Ca^2+^ sensors, which translate the signal into phosphorylation cascades and transcriptional activation of defense genes, positioning Ca^2+^ signaling as a central hub linking pathogen perception to immune responses in olive (Gómez-Lama Cabanás *et al*., 2015). Whether specific calcium signatures contribute to pathogen-specific defense outputs remains unknown, representing an important area for future investigations.

Following Ca^2+^-mediated signaling, *O. europaea* activates an early oxidative burst against pathogens such as *Xf*, *V. dahliae*, *Colletotrichum* spp., and *S. oleaginea*, marked by rapid ROS [mainly hydrogen peroxide (H_2_O_2_)] accumulation with pathogen- and genotype-dependent kinetics. This response involves induction of NADPH oxidases (e.g. RbohD-1) and protein kinases, and in resistant cultivars higher ROS levels support a signaling rather than a merely toxic role (Novelli *et al*., 2019). *Colletotrichum* spp. elicit a fast, strong oxidative burst prior to symptom onset, whereas *V. dahliae* induces a delayed but sustained ROS response accompanied by increased antioxidant enzymes such as superoxide dismutase and glutathione peroxidases, reflecting tight ROS homeostasis (Jiménez-Ruiz *et al*., 2017; Conti Taguali *et al*., 2025a). Transcriptomic data from *S. oleaginea* infection also indicate early induction of oxidative stress-related genes, including ankyrin repeat proteins, reinforcing ROS as central signaling mediators. Overall, ROS dynamics integrate with SA and JA pathways to regulate defense gene expression and systemic acquired resistance, shaping pathogen outcomes in olive (López-Moral *et al*., 2022). The ability to maintain ROS homeostasis may represent a critical determinant of quantitative resistance, since excessive ROS accumulation may cause cellular damage, whereas controlled ROS production supports defense signaling and immune activation.

Genomic annotation in *O. europaea* has identified mitogen-activated protein kinase (MAPK) genes, including a *MAPK15*-like sequence in var. *sylvestris* containing the conserved TDY motif. In cv. Koroneiki infected with *S. oleaginea*, transcriptomic data show co-expression of serine/threonine receptor kinases and downstream transcription factors (WRKY, MYB, and AP2/ERF), consistent with activation of a putative MAPK cascade downstream of PRR signaling (Marchese *et al*., 2023b; Fig. 1B). More broadly, pathogen infection induces RLKs, cell wall-associated kinases, MAPK components, and hormone-responsive transcription factors in olive (Giampetruzzi *et al*., 2016; Jiménez-Ruiz *et al*., 2017; Estudillo *et al*., 2025), supporting a functional PTI-like signaling framework. However, the complete MAPKKK→MAPKK→MAPK cascade and its direct targets have not been biochemically validated in olive, so this pathway remains inferred rather than experimentally confirmed.

### Hormonal regulation of olive defense responses

Defense responses of olive against vascular pathogens and herbivores involve the coordinated action of SA, JA, and ET signaling pathways, each playing a distinct temporal role (Fig. 1C). During *V. dahliae* infection, SA accumulates rapidly and induces PR genes (*PR1*, *PR2*, and *PR5*) together with transcription factors such as WRKY5. Resistant cultivars show stronger and earlier activation, which correlate with reduced disease severity (Gharbi *et al*., 2016; Leyva-Pérez *et al*., 2018; Cardoni *et al*., 2022; López-Moral *et al*., 2022). As infection progresses, the levels of JA and its bioactive conjugate, JA-Ile increase, particularly in elicited plants, reflecting activation of JA-dependent defenses linked to systemic resistance and secondary metabolite production (López-Moral *et al*., 2022). Herbivory by *Bactrocera oleae* further stimulates this defence network through a rapid ET burst, alterations in 12-oxo-phytodienoic acid (OPDA) and other jasmonate intermediates, and modulation of defense-related transcription factors, highlighting the contribution of both ET and JA signaling pathways (Alagna *et al*., 2016). Overall, olive defense follows a phased hormonal pattern characterized by early SA dominance during pathogen establishment and subsequent activation of JA/ET-dependent pathways during disease progression (Fig. 1C). Although SA-, JA-, and ET-mediated defense responses are increasingly recognized in olive, how their interactions regulate cultivar-dependent resistance remains largely unresolved.

### Metabolic and structural defense outputs

Immune activation in olive triggers targeted metabolic and structural reprogramming (Fig. 1D). Infection by *Alternaria alternata* induces the expression of genes involved in flavonoid and phenylpropanoid biosynthesis, resulting in the accumulation of defensive secondary metabolites (T. Wang *et al*., 2024). Likewise, resistant cultivars challenged with *S. oleaginea* show increased expression of pathogenesis-related proteins and resistance (R) gene analogs (RGAs), reflecting a substantial transcriptional remodeling of defense pathways (Marchese *et al*., 2023b). Herbivory by *B. oleae* induces hormone signaling, oxidative stress responses, and secondary metabolite biosynthesis, thereby linking immune perception to downstream metabolic and structural responses (Grasso *et al*., 2017). These responses are coordinated by hormonal-regulated networks regulating ROS production, phenylpropanoid metabolism, and cell wall reinforcement. Structural defenses, including lignification and phenolic accumulation, contribute to restricting pathogen colonization and vascular spread. Omics evidence for activation of lignin biosynthetic pathways is supported by experimental demonstrations of pathogen-induced lignification and vascular reinforcement in olive tissues (Gharbi *et al*., 2017; Jiménez-Ruiz *et al*., 2017; Novelli *et al*., 2019; Cardoni *et al*., 2022), supporting the model depicted in Fig. 1D.

### Effector-triggered immunity

ETI (Fig. 1E) in *O. europaea* is proposed to rely on NLRs (nucleotide-binding domain- and LRR-containing receptors) capable of recognizing pathogen effectors and activating strong defense responses, such as hypersensitive cell death and systemic immune signaling (Giampetruzzi *et al*., 2016; Estudillo *et al*., 2025). Genomic data indicate an expanded repertoire of NLR-like genes, supporting the existence of a putative ETI system; however, direct avirulence (Avr)–resistance (R) gene interactions remain largely unverified.

In bacterial infections, *P. savastanoi* pv. *savastanoi* effectors (e.g. HopAZ1 and HopBL1) appear to suppress or evade ETI, while in oomycetes *Phytophthora oleae* secretes RxLR, CRN, elicitins, and necrosis-inducing proteins that are typically ETI-activating in other plants, though no receptor recognition has been demonstrated in olive (Buonaurio *et al*., 2015; Rapicavoli *et al*., 2018a, b; Uceda-Campos *et al*., 2022; Conti Taguali *et al*., 2025b). Similarly, although the *V. dahliae* effector Ave1 triggers Ve1-mediated ETI in other species, neither functional recognition nor corresponding olive R genes have been identified despite the presence of Ave1 homologs. Overall, the ETI model in Fig. 1E represents a conserved conceptual framework rather than a fully validated system in olive, reflecting limitations of a non-model woody species where genetic tools are scarce and transcriptomic studies have mainly identified candidate effectors rather than confirmed Avr–R interactions (De Jonge *et al*., 2012; Estudillo *et al*., 2025).

## Role of multilayered omics

Most olive studies have relied on single-omics approaches; however, a shift toward multi-omics is necessary because plant defense and tolerance mechanisms involve complex interactions among genes, metabolites, and microbial communities that cannot be captured by a single layer. Integrating genomic, transcriptomic, proteomic, and metabolomic data enables a more complete understanding of coordinated defense mechanisms (Table 1), including hormone signaling and metabolic reprogramming, and supports improved disease management strategies. In this context, epigenetic regulation may also play a role in stress-induced gene expression, although it remains unexplored in olive.

**Table 1.** A detailed summary of omics studies addressing olive defense mechanisms against biotic stress, including the type of omics applied, the associated biotic stress agents, and the important molecular, biochemical, or physiological outcomes related to plant resistance

Publication	Omics category	Biotic stress agents	Outcomes
Estudillo *et al*. (2025)	Genomics	*Spilocaea oleaginea*	Identified multiple olive genes homologous to apple scab resistance (Rvi) genes, suggesting that olives may possess similar genetic mechanisms for disease resistance.
Serrano *et al*. (2020)	Genomics	*Verticillium dahliae*	Identified nucleotide diversity in candidate resistance genes that are potentially involved in olive defense against *V. dahliae*. TLP1 (thaumatin-like protein) and PFN2 were highlighted for their potential roles in pathogen recognition and disease resistance responses.
Cardoni *et al*. (2023)	Metabolomics	*Verticillium dahliae*	Showed that tolerant cultivars have higher basal levels of secoiridoids (e.g. oleuropein-related compounds), whereas susceptible cultivars accumulate compounds such as verbascoside and lignan derivatives. Demonstrated that infection induces only minor and transient metabolic changes, indicating that olive tolerance is primarily driven by pre-formed phenolic and triterpenic defenses rather than extensive inducible metabolomic reprogramming.
Conti Taguali *et al*. (2025a)	Metabolomics	*Colletotrichum* spp.	Olive response to *Colletotrichum* infection is strongly cultivar dependent, with ‘Leccino’ being the most tolerant and ‘Ottobratica’ the most susceptible. Infection triggered distinct physiological and biochemical changes, including oxidative stress and metabolic shifts.
Di Masi *et al*. (2022)	Metabolomics	*Xylella fastidiosa*	Modulation of phenolic and secondary metabolites, together with shifts in oxidative stress-related and lipid compounds, reflects coordinated antimicrobial, ROS-driven, and membrane-related defense responses in olive.
Girelli *et al*. (2021)	Metabolomics	*Xylella fastidiosa*	Tolerant and susceptible olive cultivars show distinct metabolomic responses to *Xf* infection, with tolerant cultivars exhibiting increased accumulation of phenolic and secondary metabolites linked to antimicrobial defense and resistance. Metabolomic changes involving stress-related pathways, including oxidative stress and lipid metabolism, can serve as indicators of defense status and tolerance for identifying resistant cultivars.
Jlilat *et al*. (2021)	Metabolomics	*Xylella fastidiosa*	An enhanced accumulation of phenolic compounds, together with alterations in oxidative stress-related metabolites and lipid and secondary metabolism, reflects integrated antimicrobial, ROS-driven, and structural defense responses to infection.
Riolo *et al*. (2023)	Metabolomics	*Colletotrichum*	The study identified 45 secondary metabolites produced by four *Colletotrichum* species during infection of olive, including fatty acids, phenolics, pyrones, sterols, and terpenes. It showed that metabolite accumulation increases during infection, peaking at later stages and correlating with disease severity.
Cardoni and Mercado-Blanco (2023)	Microbiomics	*Verticillium dahliae*, *Xylella fastidiosa*, *Pseudomonas savastanoi*, *Colletotrichum*, *Spilocaea oleaginea*, *Phytophthora*, *Meloidogyne*, *Pratylenchus*, *Bactrocera oleae*, and *Prays oleae*	Olive stress tolerance depends on the holobiont system, where plant performance under biotic stress is shaped by interactions between the host, root traits, and associated microbiome, rather than plant genetics alone. Disease suppression is linked to beneficial microbiome recruitment, where microbes such as *Pseudomonas* and *Paenibacillus* spp. help suppress pathogens and are influenced by cultivar-dependent root traits and constitutive chemical defenses.
Fernández-González *et al*. (2020)	Microbiomics	*Verticillium dahliae*	‘Frantoio’ (tolerant) and ‘Picual’ (susceptible) showed similar baseline microbial diversity but differed in composition, with ‘Frantoio’ enriched in beneficial taxa and maintaining greater microbiome stability after *V. dahliae* infection, whereas ‘Picual’ exhibited stronger microbial disruption. Infection also induced more connected and stable microbial networks in ‘Frantoio’, while ‘Picual’ showed fragmented networks and stronger shifts in arbuscular mycorrhizal fungal distribution.
Fernández-González *et al*. (2025)	Microbiomics and transcriptomics	*Verticillium dahliae*	Found enrichment of defense- and metabolism-related biological processes in tolerant cultivars, including carbohydrate metabolism, amino acid transport, transcription regulation, oxidoreductase activity, and immune response pathways, indicating coordinated metabolic–defense reprogramming. Identified keystone microbial taxa associated with tolerance, particularly bacterial genera such as *Actinophytocola*, *Kibdelosporangium*, and *Nocardia*, which were significantly enriched in tolerant olive genotypes.
Vergine *et al*. (2024)	Microbiomics	*Xylella fastidiosa*	Asymptomatic or low symptomatic olive genotypes exhibited a distinct endophytic microbiome compared with highly symptomatic plants under the same *Xf* pressure, indicating strong genotype-driven selection of microbial communities. Tolerant or low symptomatic olive genotypes were enriched in beneficial bacterial genera such as *Burkholderia*, *Bacillus*, *Sphingomonas*, and *Hymenobacter*, which are commonly associated with plant growth promotion and pathogen suppression.
Corrado *et al*. (2012)	Proteomics	*Bactrocera oleae*	Olive fruits respond to *B. oleae* infestation through complex molecular changes, including activation of stress responses, phytohormone signaling, and defense-related pathways. Identified specific defense-related genes and proteins, such as chitinases and trypsin inhibitors.
C. Wang *et al*. (2024)	Transcriptomics and metabolomics	*Alternaria alternata*	Flavonoid biosynthesis genes, including chalcone synthase (*CHS*), cinnamate 4-hydroxylase (*C4H*), and flavanone 3-hydroxylase (*F3H*), were strongly up-regulated in resistant olive cultivars, indicating their direct role in defense against *A. alternata*.
Amoia *et al*. (2025)	Transcriptomics	*Xylella fastidiosa*	The bacteriocin gene *cvaC-1* is highly overexpressed during infection, making it a reliable molecular marker for detecting active growth of *Xf* in olive. Variation in cvaC-1/cvaC-2 ratio across strains indicates strain-specific infection dynamics.
Arnholdt-Schmitt *et al*. (2024)	Transcriptomics	*Xylella fastidiosa*	Tolerance to *Xf* is linked to metabolic adjustments, particularly enhanced aerobic fermentation, phenolic metabolism, and cyanide-mediated regulation of respiration. Increased expression of the alternative oxidase (*AOX*) gene family as a key marker of resilience.
Balan *et al*. (2025)	Transcriptomics	*Xylella fastidiosa*	Immune regulators (BAK1, WRKYs) form interconnected defense hubs which can be the targets for broad-spectrum resistance in plants including olive. Plant defense against *Xf* involves hormonal signaling (JA, SA, ABA) and metabolic pathways that enhance stress response and antimicrobial compound production.
Giampetruzzi *et al*. (2016)	Transcriptomics	*Xylella fastidiosa*	*Xf* infection triggers distinct transcriptomic responses in olive cultivars, with the tolerant ‘Leccino’ activating stronger defense mechanisms than the susceptible ‘Ogliarola salentina’. Showed that enhanced expression of receptor-like kinases and cell wall-related genes in ‘Leccino’ contributes to its lower pathogen levels and intrinsic tolerance.
Gómez-Lama Cabanás *et al*. (2015)	Transcriptomics	*Verticillium dahliae*	*V. dahliae* induces extensive systemic transcriptomic changes in the tolerant olive cultivar ‘Frantoio’, involving many genes related to defense responses. Identified specific genes, such as *GRAS1* and *DRR2*, whose expression patterns are associated with tolerance.
Grasso *et al*. (2017)	Transcriptomics	*Bactrocera oleae*	Tolerant olive cultivar shows widespread activation of genes involved in oxidative stress responses, hormone signaling, and secondary metabolism. Genes affecting cellular structure and primary metabolism are differentially regulated as a result of the induced defense.
Jiménez-Ruiz *et al*. (2019)	Transcriptomics	*Verticillium dahliae*	Pathogen-activated genes related to virulence, pathogenicity, niche adaptation, and microsclerotia development mainly in ‘Picual’, while these genes remain largely unexpressed in ‘Frantoio’.
Jiménez-Ruiz *et al*. (2017)	Transcriptomics	*Verticillium dahliae*	Coordinated transcriptomic changes happened during infection, with genes related to proteolysis, protein folding, and biosynthesis. Revealed a temporal defense pattern in which ROS responses are activated first in the pathogen and later in the olive roots.
La Notte *et al*. (2024)	Transcriptomics	*Xylella fastidiosa*	Identified large-scale transcriptomic changes in resistant genotypes, highlighting key pathways such as photosynthesis, cell wall, and metabolism.
Leyva-Pérez *et al*. (2018)	Transcriptomics	*Verticillium dahliae*	Identified key defense-related genes, including those involved in ROS response, lignification, and pathogen recognition, that are differentially expressed and probably contribute to the tolerant phenotype. Genes related to pathogen recognition (e.g. *BAK1*, *NHL1*) and defense signaling are more effectively regulated in the tolerant cultivar.
Marchese *et al*. (2023b)	Transcriptomics	*Spilocaea oleaginea*	Cultivar ‘Koroneiki’ mounts a strong and early defense response against *S. oleaginea*, characterized by extensive hormonal crosstalk involving ABA, ethylene, and jasmonate. Results highlight enhanced cell wall biosynthesis and remodeling, including lignin formation.
Mascuñano *et al*. (2025)	Transcriptomics	*Verticillium dahliae*	Resistance to *V. dahliae* in wild olive clone AC18 is associated with constitutively high lignin levels, which act as a pre-formed physical defense barrier. Highlights the role of cell wall strengthening and structural defense mechanisms as a key factor in resistance to Verticillium wilt.
Martí *et al*. (2020)	Metatranscriptomics	*Verticillium dahliae*	Infection triggered microbiome-driven defense modulation, where shifts in microbial gene expression influence the overall defense outcome. Co-existence of biotrophic and necrotrophic microorganisms requires the plant to coordinate multiple defence pathways, probably involving both salicylic acid- and jasmonate-related responses.

### Genomic resources for olive defense studies

Genomic resources provide the essential foundation for understanding the molecular basis of biotic stress responses in olive, aiding the identification of genetic determinants underlying resistance, tolerance, and adaptation. Whole-genome scanning has expanded this knowledge by revealing extensive genomic variation across Mediterranean germplasm and identifying candidate genes associated with key traits (Bazakos *et al*., 2023), providing a framework to connect genetic diversity with stress responses. In parallel, olive exhibits a distinctive genome structure shaped by the expansion of tandem repeats rather than typical transposable element dynamics, suggesting high genomic plasticity and regulatory complexity (Mascagni *et al*., 2022). Together, these advances support omics approaches to dissect defense mechanisms and improve olive resilience to biotic stresses.

#### Olive genomes

Advances in genomic resources have transformed the study of disease resistance in olive, providing a foundation for understanding the genetic basis of host defense. Sequencing and assembly of the olive genome have generated high-quality reference maps that enable the identification of resistance-associated loci and candidate genes. These genomic frameworks complement transcriptomic analyses by facilitating the discovery of RGAs and quantitative trait loci (QTLs), while genome-wide association studies (GWAS) and linkage mapping are increasingly used to link genetic variation with pathogen resistance. Collectively, these resources offer powerful tools for breeding programs, allowing marker-assisted and genomic selection in a species with long juvenile phases and complex reproductive biology, and enabling comparative analyses that explore diversity among cultivars and wild relatives to understand the evolutionary dynamics of defense-related traits.

The growing availability of *Olea* genome assemblies (Table 2) has provided an essential foundation for dissecting the genetic basis of disease resistance. Early draft genomes, such as the cv. Farga, offered scaffold-level references anchored to linkage groups, enabling the initial identification of RGAs and candidate loci (Cruz *et al*., 2016). Concurrently, the draft assembly of *O. europaea* subsp. *sylvestris* regarded as the wild ancestor of cultivated olive, provided a partially anchored genome that facilitated initial comparative and domestication studies (Unver *et al*., 2017). The scaffold-level PacBio assembly of cv. Picual, further improved through Illumina-based polishing, increased sequence continuity relative to short-read drafts and enhanced the characterization of repetitive regions and gene families, including those implicated in stress responses (Jiménez-Ruiz *et al*., 2020). Subsequent integration of long-read sequencing technologies with Hi-C scaffolding substantially enhanced genome contiguity, as exemplified by the chromosome-level assembly of cv. Arbequina, which resolved sequences into 23 pseudochromosomes and significantly improved structural annotation and gene model accuracy (Rao *et al*., 2021).

**Table 2.** Summary of major *Olea europaea* genome assemblies and their assembly characteristics

Assembly/name	Assembly level	Approx. size	References
*O. europaea* var. *europaea* cv. Farga (Oe9)	Chromosome-anchored draft	∼1.32 Gb	Cruz *et al*. (2016)
*O. europaea* subsp. *sylvestris* (Oe451_v1)	Draft assembly, partially anchored	∼1.48 Gb	Unver *et al*. (2017)
*O. europaea* cv. Picual (Oleur061)	Draft assembly—scaffold-level (PacBio+Illumina reads)	∼1.68 Gb	Jiménez-Ruiz *et al*. (2020)
*O. europaea* cv. Arbequina (Oe_Rao)	Chromosome-level (Hi-C)	∼1.30 Gb	Rao *et al*. (2021)
*O. europaea* cv. (telomere-to-telomere; T2T) ‘Leccino’	Chromosome-level gap-free T2Tassembly	∼1.28 Gb	Lv *et al*. (2024)
*O. europaea* cv. Leccino (2025 PacBio)	Contig level (HiFi)	∼1.43 Gb	Sebastiani *et al*. (2025)
*O. europaea* cv. Frantoio (2025 PacBio)	Contig level (HiFi)	∼1.18 Gb	Sebastiani *et al*. (2025)

The recent gapless genome assembly of the tolerant cv. Leccino represents a major advance in olive genomics by improving the resolution of complex regions, including resistance gene clusters and regulatory elements, thereby supporting studies on quantitative resistance and genomic selection against pathogens such as *Xf* (Lv *et al*., 2024). In parallel, highly complete PacBio HiFi-based assemblies of cultivars such as Leccino and Frantoio further enhanced genomic resources for comparative genomics, GWAS, and marker-assisted identification of pathogen resistance loci in olive (Sebastiani *et al*., 2025).

QTL analysis is a valuable approach for dissecting complex disease resistance traits by linking phenotypic variation with molecular markers to identify loci underlying quantitative resistance (Young, 1996). Although resistance-focused QTL studies in *O. europaea* are still limited, studies in *Malus domestica* and *Prunus dulcis* demonstrate its potential (Le Roux *et al*., 2010; Wöhner *et al*., 2014; Calle *et al*., 2025). In olive, QTL analyses have mainly targeted agronomic traits such as flowering, oil composition, frost tolerance, and vigor (Ben Sadok *et al*., 2013; Kaya *et al*., 2024; Çetin *et al*., 2025; Granata *et al*., 2025; Lamoumni *et al*., 2025). Candidate-gene studies also identified single nucleotide polymorphisms (SNPs) and insertions/deletions (InDels) in defense-related genes (*TLP1*, *PFN2*, *DRR2*, and *COMT*) associated with *V. dahliae* responses (Serrano *et al*., 2020). However, markers for resistance to major pathogens such as *Xf* and *Colletotrichum* spp. are still lacking, emphasizing the need for dense SNP genotyping, standardized phenotyping, and genomic selection strategies.

#### Resistance gene analogs and resistance repertoires in olive

High-quality genome sequences and transcriptomic resources in *O. europaea* have enabled *in silico* annotation of immune-related gene families, including NLR-like sequences and RLK families, which are frequently differentially expressed under stress and are implicated in pathogen perception and defense activation (Lagiotis *et al*., 2024). Although no functional R-genes have yet been experimentally validated against major pathogens such as *V. dahliae*, *Xf*, or *Colletotrichum* spp., the presence of annotated receptor and NLR gene families provides a foundation for future studies (Parvez *et al*., 2025). Integrating RGA annotation with expression profiling, GWAS, and pan-genomic analyses may help identify loci underlying quantitative disease resistance in olive.

### Transcriptomic approaches

Traditional plant pathology and conventional molecular methods, such as microscopy, culture-based pathogen identification, targeted gene expression analyses (e.g. qPCR), and biochemical assays, have long been foundational in studying olive diseases by revealing visible symptoms, pathogen presence, and specific host responses (Campos *et al*., 2019; Gharsallah *et al*., 2020; Conti Taguali *et al*., 2025a). However, traditional approaches often focus on single pathogens or pre-defined host genes, providing only partial insight into the complex molecular interactions occurring during infection. In contrast, transcriptomic technologies enable unbiased genome-wide analysis of gene expression in both host and pathogen simultaneously, offering a broader and more precise understanding of disease processes at cellular and tissue-specific levels that are difficult to capture using conventional methods (Jiménez-Ruiz *et al*., 2019).

The identification of differentially expressed genes in RNA-seq studies is particularly valuable for gaining an understanding of complex molecular pathways, such as those involved in pathogen recognition and defense activation. Comparative transcriptomic studies of *O. europaea* infected with *V. dahliae* revealed cultivar-dependent responses, with the tolerant cv. Frantoio showing stronger expression of defense-related genes, including *BAK1*, *NHL1*, dirigent-like proteins, and ROS-associated pathways, whereas the susceptible cv. Picual exhibited higher expression of fungal virulence- and signaling-related genes (Leyva-Pérez *et al*., 2018; Jiménez-Ruiz *et al*., 2019). Similarly, dual RNA-seq analyses of *Xf*-infected olive tissues identified strong *in planta* overexpression of bacterial bacteriocin-related genes, including *cvaC-1*, *cvaC-2*, and Blp family class II bacteriocins (Amoia *et al*., 2025), implicated in bacterial competition, colonization, and host interaction.

Collectively, these findings demonstrate that transcriptomics not only identifies candidate defense genes but also captures the temporal and regulatory complexity of olive–pathogen interactions. Importantly, the choice of transcriptomic platform critically determines the depth, sensitivity, and interpretative resolution of these analyses.

Building on RNA-seq studies of infection-induced gene expression, higher-resolution approaches such as single-cell RNA sequencing (scRNA-seq) and spatial transcriptomics could further resolve host responses at single-cell and spatial levels, particularly in spatially structured infections such as *Xf* colonization of xylem or *S. oleaginea* infection of leaf mesophyll. However, these technologies have not yet been applied to olive–pathogen systems, representing a key methodological gap and a promising avenue to uncover cell-type-specific mechanisms of quantitative resistance.

#### Microarrays

Microarrays are hybridization-based tools that use pre-defined probes to detect known transcripts; while limited to previously characterized genes, they remain useful for expression analysis in resource-limited settings due to their lower cost and minimal computational demands.

In *O. europaea*, a custom microarray comprising ∼60 000 probes derived from 454-based unigene assemblies and implemented on a CustomArray platform was employed to profile transcriptional responses in drupes challenged by *B. oleae*, allowing a direct comparison between tolerant and susceptible cultivars. Differences in transcriptional profiles between cultivars were associated with less than half the number of genes in the infestation response of the tolerant genotype alone, yet a majority of these genes were up-regulated, supporting the idea that differences in tolerance between the two varieties are driven more by the targeted induction of key genes rather than by widespread changes in gene expression (Grasso *et al*., 2017). However, microarrays were not widely adopted for pathogen response studies in olive, largely because early research was constrained by incomplete genomic resources, which hindered reliable probe design. Furthermore, interactions with pathogens such as *Xf* or *V. dahliae* require detection of novel transcripts and low-abundance genes, tasks for which microarrays are inherently limited due to restricted dynamic range and cross-hybridization issues. As genomic resources improved, RNA-seq rapidly became the preferred platform for unbiased, high-resolution transcriptome profiling (Rai *et al*., 2018).

#### RNA sequencing

Unlike microarrays, RNA-seq does not rely on pre-defined probes but directly sequences cDNA fragments, enabling detection of rare transcripts, alternative splice variants, and non-coding RNAs (Sekhon *et al*., 2013). Consequently, most recent studies on olive–pathogen interactions have adopted RNA-seq.

RNA-seq has been central to understanding olive tolerance to *Xf* subsp. *pauca*. Giampetruzzi *et al*. (2016) showed extensive transcriptional reprogramming involving RLKs, hormone signaling, and cell wall modification genes, while Arnholdt-Schmitt *et al*. (2024) linked tolerance to acetaldehyde production, cyanide-resistant respiration, and regulation of the alternative oxidase (AOX) pathway, highlighting mitochondrial redox balance. Structural defenses also contribute, with increased lignin deposition and elevated quinic acid, along with up-regulation of cinnamoyl-CoA reductase and polyphenol oxidase in the tolerant cv. Leccino (Sabella *et al*., 2018). Field studies further confirmed heritable tolerance traits in Leccino progeny under high infection pressure (La Notte *et al*., 2024), while pathogen-side transcriptomics revealed bacterial induction of bacteriocins during infection, indicating host–pathogen transcriptional interplay (Amoia *et al*., 2025). Collectively, olive tolerance to *Xf* reflects integrated regulation of immune signaling, vascular reinforcement, redox homeostasis, and mitochondrial respiratory plasticity.

Integrative transcriptomic and protein network analyses indicate that tolerance to *Xf* in *O. europaea* involves coordinated regulation of RLK genes such as *BAK1*, MAPK cascades, WRKY transcription factors, lipid remodeling enzymes, and carbohydrate metabolism pathways (Balan *et al*., 2025). Tolerant cultivars exhibit moderated and sustained transcriptional responses associated with vascular homeostasis and metabolic balance. Similarly, RNA-seq studies of *V. dahliae* identified early activation of pathogen perception, MAPK signaling, phenylpropanoid metabolism, and oxidative stress pathways (Jiménez-Ruiz *et al*., 2017), while tolerant cv. Frantoio displayed stronger lignification and JA/ET signaling responses (Leyva-Pérez *et al*., 2018). Transcriptomic plasticity also varied with cultivar susceptibility (Jiménez-Ruiz *et al*., 2019). In *S. oleaginea* interactions, resistant genotypes showed enhanced induction of pathogen recognition, phenylpropanoid/flavonoid biosynthesis, and ROS detoxification pathways (Marchese *et al*., 2023b).

#### Metatranscriptomics

Building on bulk RNA-seq, metatranscriptomics enables simultaneous profiling of gene expression from the host and associated microbial communities. Reanalysis of RNA-seq data from *O. europaea* roots infected with *V. dahliae* revealed a temporal shift from predominantly host-derived transcripts to a diverse microbial transcript pool, reflecting increasing activity of fungi, bacteria, and protists during infection. These findings demonstrated that Verticillium wilt is not merely a binary host–pathogen interaction but a dynamic polymicrobial process shaped by rhizosphere community activity, highlighting the value of metatranscriptomics for resolving ecological complexity inaccessible to single-organism approaches (Martí *et al*., 2020).

### Metabolomics

Metabolomics provides a direct functional readout of plant defense activation by capturing changes in primary and secondary metabolites during pathogen challenge. In olive, metabolomic investigations of biotic stress responses are limited but are beginning to reveal infection-associated biochemical reprogramming, particularly in fruit tissues affected by anthracnose.

In olive, resistance to *V. dahliae* is primarily associated with constitutive root metabolite profiles rather than extensive induced changes. Tolerant cultivars display higher basal levels of secoiridoids, particularly oleuropein derivatives and elenolic acid glucosides, while susceptible cultivars accumulate compounds such as verbascoside and methoxypinoresinol glucoside. These phenolic and triterpenic metabolites contribute to defense through antifungal and antioxidant activities, limiting pathogen establishment at the root level. Notably, infection triggers only minor and transient metabolic shifts, indicating that resistance relies mainly on pre-formed chemical defenses and rapid early responses, rather than large-scale metabolomic reprogramming (Cardoni *et al*., 2023).

Recent metabolomics studies of *O. europaea–Colletotrichum* interactions reveal coordinated host and pathogen metabolic responses during infection. In infected olive drupes, reductions in Chl *a*, Chl *b*, and carotenoids indicate impaired photosynthesis and pigment degradation, while increased H_2_O_2_, malondialdehyde (MDA), and altered soluble sugar levels reflect oxidative stress, lipid peroxidation, and shifts in primary metabolism (Conti Taguali *et al*., 2025a). Complementary metabolomic profiling of *Colletotrichum acutatum*, *C. gloeosporioides*, *C. godetiae*, and *C. karsti* identified 45 fungal metabolites, including fatty acids, phenolics, pyrones, sterols, and terpenoid-related compounds (Riolo *et al*., 2023). Infection was associated with accumulation of polyketide-derived toxins such as pyrenocine B, which increased during disease progression and peaked at late infection stages. Together, these findings indicate that olive anthracnose involves both host metabolic disruption and pathogen-derived secondary metabolite production that collectively shape disease progression.

Metabolomic studies of *Xf*-infected olive plants have shown accumulation of sugars such as mannitol and sucrose, along with shifts in organic acids, reflecting adjustments in primary metabolism to cope with stress (Jlilat *et al*., 2021). Secondary metabolites, including oleuropein and several flavonoids, are often altered, indicating modulation of defense-related pathways. Stress-associated compounds such as pyridoxine, ABA, and certain fatty acids are also increased, suggesting the activation of stress and defense response pathways (Di Masi *et al*., 2022). Comparisons between tolerant and susceptible cultivars in responses to *Xf* infection show differences in flavonoid, phenolic, and sugar metabolism, highlighting cultivar-specific responses and the influence of both genetic background and experimental treatments on host metabolomic profiles (Girelli *et al*., 2021).

Across olive–pathogen interactions, phenolic and primary metabolism are consistently involved, but responses differ by pathogen lifestyle. In *V. dahliae*, resistance relies mainly on constitutive secoiridoids (e.g. oleuropein derivatives) with limited metabolic reprogramming. In contrast, *Colletotrichum* infection induces strong oxidative stress, pigment degradation, and sugar metabolism shifts, alongside fungal secondary metabolites linked to disease progression. *Xf* also triggers broad changes in sugars, organic acids, and phenolics (including oleuropein and flavonoids) with cultivar-dependent variation. Overall, olive defense ranges from pre-formed chemical resistance to extensive inducible metabolic reprogramming, depending on the pathogen.

### Proteomics

Proteomic investigations of olive responses to biotic stress remain comparatively underdeveloped relative to transcriptomic studies, yet they are uniquely positioned to bridge the gap between gene expression and functional phenotype. Unlike transcript abundance, protein accumulation, post-translational modification, and subcellular redistribution more directly reflect the biochemical state of immune signaling networks and metabolic fluxes. One of the earliest proteomic studies in olive focused on herbivory, particularly infestation by the olive fruit fly, a major constraint on drupe quality and yield. Using two-dimensional electrophoresis coupled with nanoLC-ESI-LIT-MS/MS, Corrado *et al*. (2012) identified proteins involved in stress signaling, phytohormone crosstalk, transcriptional regulation, redox balance, and primary carbon metabolism, revealing that olive fruits undergo extensive proteome remodeling in response to biotic challenge. These findings underscore that olive defense extends beyond canonical immune pathways and involves coordinated metabolic and signaling reprogramming. Methodological advances in protein extraction and MS optimization (Capriotti *et al*., 2013) have since improved analytical robustness, enabling higher-resolution profiling of olive tissues rich in phenolics and lipids. However, direct proteomic characterization of olive responses to major vascular pathogens such as *V. dahliae* and *Xf* remains largely unexplored. This represents a critical gap, particularly given that vascular pathogens perturb xylem integrity, systemic signaling, and long-distance metabolic coordination, processes that are not fully captured by transcriptomic data alone. Despite extensive transcriptomic characterization of olive immunity, the scarcity of large-scale proteomic datasets limits the connection between gene expression and functional resistance Given that protein activity is extensively shaped by phosphorylation, redox regulation, and subcellular dynamics, advanced proteomics approaches will be crucial for the identification of molecular regulators, connecting immune signaling with resistance in olive. Integrating quantitative, phospho-, redox-, and spatial resolved vascular proteomics, therefore, represents a key priority for future research.

### Microbiomics

Although olive defense has been studied primarily from a host-centric perspective, olive trees function as holobionts whose health, stress resilience, and productivity are strongly shaped by associated microbial communities. Consequently, stress responses should be viewed at the whole-system level, integrating host physiology with microbiome dynamics. Olive-associated microbiota influence disease outcomes through competitive interactions, network dynamics, and biocontrol functions, while biotic stresses can disrupt these consortia. Conversely, specific microbial taxa can enhance host defense and resilience, highlighting the potential of microbiome-informed strategies for sustainable disease management (Cardoni and Mercado-Blanco, 2023).

*Xf* infection responses in olive are strongly associated with differences in endophytic microbiome composition across genotypes, indicating that tolerance is not solely genotype driven but closely linked to specific microbial assemblages. Tolerant or asymptomatic cultivars harbor a more structured endosphere community enriched in bacterial taxa such as *Pseudomonas*, *Sphingomonas*, *Methylobacterium*, and *Bacillus/Paenibacillus*, widely recognized for plant growth promotion and pathogen antagonism. These genotypes also show enrichment of fungal endophytes such as *Cladosporium* and *Alternaria*, whereas susceptible plants exhibit more dysbiotic fungal profiles dominated by stress-associated assemblages (Vergine *et al*., 2024).

Olive resistance or reduced disease severity associated with *V. dahliae* is linked to coordinated host–microbiome interactions within the holobiont, integrating plant immune regulation and below-ground microbial network structure. Resistant cultivars exhibit up-regulation of genes involved in immune signaling and defense activation, including transcription factors (MYB, bHLH), receptor-like kinases, ET-responsive factors, and pathogenesis-related proteins such as β-1,3-glucanases and thaumatin-like proteins, along with genes related to oxidative stress and secondary metabolism. These host responses are closely associated with a structured root microbiome enriched in beneficial taxa, particularly Proteobacteria and Actinobacteria, and keystone genera such as *Actinophytocola*, *Kibdelosporangium*, and *Nocardia*, which correlate with defense gene expression and negatively associate with *Verticillium* spp., suggesting a role in pathogen suppression. In contrast, susceptible cultivars display more disrupted microbial networks, lower resilience, and incorporation of *V. dahliae* into the community, often accompanied by positively connected fungal hubs (Fernández-González *et al*., 2020, 2025).

Collectively, these findings emphasize that olive defense depends on interactions between host genetic responses and associated microbial communities. Integrating microbiomics with host-centered approaches is therefore essential for understanding disease outcomes and developing resilient, sustainable disease management strategies in olive.

## Discussion

Given that fully integrated multi-omics studies in olive are still scarce, it is essential to consider a set of strongly interconnected works that collectively represent a stepwise multi-omics exploration of the system. Cardoni *et al*. (2022) analyzed root defense responses to Verticillium wilt using targeted gene expression and physiological assays, which was complemented by the same authors (Cardoni *et al*., 2023) through metabolomics profiling of phenolic and triterpenic compounds linked to resistance. These findings were then synthesized by Cardoni and Mercado-Blanco (2023) into a broader framework integrating transcriptomics, metabolomics, and microbiomics to conceptualize olive resilience. Similarly, Fernández-González *et al*. (2020) showed cultivar-dependent differences in below-ground microbial networks and keystone taxa, followed by their further study (Fernández-González *et al*., 2021), which integrated endophytic microbiome data with host transcriptomic responses. The study by Fernández-González *et al*. (2025) further consolidated these approaches by jointly identifying key host genes and microbial taxa associated with tolerance, moving toward a unified holobiont-level interpretation of disease resistance.

Plant immunity in olive is a multilayered system that broadly follows the canonical PTI–ETI framework established in *Arabidopsis thaliana*, but with key differences in functional validation. At the basal level, olive relies more heavily than Arabidopsis on constitutive defenses, including lignified cell walls and phenolic-rich compounds such as oleuropein derivatives, which function as pre-formed structural and antimicrobial barriers against pathogens such as *V. dahliae* (Jones and Dangl, 2006; Luvisi *et al*., 2017a; Saponari *et al*., 2019; Marchese *et al*., 2023b). In both olive and Arabidopsis, PTI is initiated by RLKs; however, while receptors such as FLS2 and EFR are functionally validated in Arabidopsis (Zipfel *et al*., 2004; Boller and Felix, 2009), RLKs in olive are mainly inferred from genomic and transcriptomic analyses without direct biochemical validation (Giampetruzzi *et al*., 2016; Estudillo *et al*., 2025). Early signaling is broadly conserved, including Ca^2+^ influx mediated by calcium-dependent protein kinases and ROS bursts generated by NADPH oxidases, which are well-established PTI outputs in Arabidopsis (Torres *et al*., 2006; Boudsocq *et al*., 2010) and similarly observed in olive–pathogen interactions (Gómez-Lama Cabanás *et al*., 2015; Novelli *et al*., 2019). Downstream signaling shows similar conceptual conservation but differing levels of mechanistic resolution. In Arabidopsis, MAPK cascades such as MPK3/MPK6 are well characterized and directly link PRR activation to transcriptional reprogramming (Asai *et al*., 2002), whereas in olive only MAPK-associated genes and transcription factors (WRKY, MYB, and AP2/ERF) have been inferred from expression studies without full pathway validation (Giampetruzzi *et al*., 2016; Marchese *et al*., 2023b). Hormonal regulation is also conserved, with SA mediating early defense against biotrophic pathogens and JA/ET coordinating later or necrotroph-associated responses in both systems (Pieterse *et al*., 2012; Gharbi *et al*., 2017; Li *et al*., 2019; López-Moral *et al*., 2022). At the output level, both species activate phenylpropanoid metabolism and structural reinforcement; however, olive relies more heavily on constitutive and long-term metabolic defenses, consistent with its perennial woody habit (Luvisi *et al*., 2017a; Cardoni *et al*., 2023), whereas Arabidopsis defense responses are predominantly inducible (Glazebrook, 2005). Finally, ETI is well established in Arabidopsis through NLR-mediated recognition of pathogen effectors (Jones and Dangl, 2006; Kourelis and van der Hoorn, 2018), while olive possesses NLR-like genes but lacks experimentally confirmed Avr–R interactions, making ETI largely conceptual rather than functionally validated (Giampetruzzi *et al*., 2016; Estudillo *et al*., 2025).

Progress in deciphering olive defense responses remains constrained by the gap between large-scale molecular discoveries and their experimental validation. This limitation largely arises from biological and technical challenges associated with *O. europaea*, including its slow growth, recalcitrance to stable transformation, and the lack of standardized genetic tools. In particular, genetic manipulation in olive is hindered by low transformation efficiency, strong genotype dependence, limited regeneration capacity, and difficulties associated with *in vitro* culture and plant recovery, collectively constraining the development of reliable transgenic systems (Palomo-Ríos *et al*., 2021). To overcome these limitations, heterologous and surrogate systems are widely used, enabling functional characterization of candidate genes in tractable model plants such as Arabidopsis. This approach has proven effective in biotic stress research, as demonstrated by the heterologous expression of cotton NLR genes conferring *Verticillium* resistance (Li *et al*., 2018) and rice WRKY transcription factors enhancing pathogen defense in Arabidopsis (Jing *et al*., 2009). In parallel, emerging functional genomics tools are expanding the experimental repertoire for woody perennial crops. Virus-induced gene silencing (VIGS) provides a rapid, transformation-independent approach for gene function analysis, as shown in citrus where silencing of *CsLOB1* reduced citrus canker susceptibility (C. Wang *et al*., 2024). Similarly, CRISPR/Cas [clustered regularly interspaced palindromic repeats (CRISPR)/CRISPR-associated protein]-mediated genome editing has enabled targeted mutagenesis in poplar, citrus, and grapevine for genes involved in development, stress responses, and pathogen resistance (Dong, 2024; Ren *et al*., 2024; S. Wang *et al*., 2024). Together, these approaches help overcome long generation times and transformation recalcitrance in woody perennials and offer promising avenues for functional validation in olive immunity research.

## Directions for future research

Although omics approaches have substantially advanced our understanding of olive responses to biotic stress, significant conceptual, technical, and translational gaps still remain. Current knowledge is still largely transcript-centric, providing only a partial view of the complex regulatory networks underlying immunity. Future progress will therefore depend on moving beyond single-omics studies toward truly integrative, systems-level multi-omics approaches that combine transcriptomic, proteomic, metabolomic, microbiomic, epigenomic, and genomic data.

A major bottleneck in olive immunity research is the scarcity of functional validation. Candidate R-genes, RLKs, transcription factors, and defense-associated metabolites have been identified, yet causal relationships remain largely untested. Future priorities include optimizing CRISPR/Cas genome editing in olive, developing heterologous and surrogate systems and VIGS-like platforms adapted to woody species, and targeting susceptibility genes to achieve durable, broad-spectrum disease resistance in olive.

Recent advances in genome sequencing have generated multiple whole-genome assemblies for olive cultivars, providing the foundation for an olive pangenome. However, a comprehensive olive pangenome has not yet been established. Future efforts should focus on constructing a high-quality olive pan-genome that includes cultivars and wild relatives to capture structural and presence–absence variation relevant to disease resistance.

In addition, epigenetic regulation of olive immunity remains largely unexplored, particularly DNA methylation, chromatin accessibility, and potential transgenerational immune memory, which may contribute to durable disease resistance. Similarly, microbiome studies are still limited and should be expanded through integrated metagenomic, metatranscriptomic, and metaproteomic approaches to identify active microbial functions and validate keystone host–microbiome interactions. Finally, integrating multi-omics data with systems biology and machine learning will support predictive models of resistance, while direct translation into breeding through marker-assisted selection, genomic selection, and pyramiding of quantitative resistance loci remains a key priority.

Overall, omics-driven research indicates that olive immunity is governed by interconnected transcriptional, hormonal, metabolic, physiological, and microbiome-associated processes rather than by single resistance genes. Although substantial progress has been achieved through genomics and transcriptomics, huge gaps remain in functional validation and in the integration of molecular information across biological scales. Therefore, future advances will depend on the combination of multi-omics datasets, functional genomics, systems biology, and predictive modeling, supported by artificial intelligence (AI), to transform descriptive knowledge into mechanistic understanding to accelerate the development of durable disease resistance in olive.

## References

[erag337-B1] Alagna F, Kallenbach M, Pompa A, De Marchis F, Rao R, Baldwin IT, Bonaventure G, Baldoni L. 2016. Olive fruits infested with olive fly larvae respond with an ethylene burst and the emission of specific volatiles. Journal of Integrative Plant Biology 58, 413–425. 10.1111/jipb.12343 25727685

[erag337-B2] Amoia SS, Saponari M, Saldarelli P, Ligorio AM, Grosso CD, Loconsole G, D’Attoma G, Boscia D, Giampetruzzi A. 2025. Preliminary transcriptomic analyses reveal in vitro and in planta overexpression of various bacteriocins in *Xylella fastidiosa*. Frontiers in Microbiology 16, 1501741. 10.3389/fmicb.2025.1501741 PMC11885251 40061858

[erag337-B3] Arnholdt-Schmitt B, Sircar D, Aziz S, Germano TA, Thiers KLL, Noceda C, Bharadwaj R, Mohanapriya G, Costa JH. 2024. Transcriptome analyses in adult olive trees indicate acetaldehyde release and cyanide-mediated respiration traits as critical for tolerance against *Xylella fastidiosa* and suggest AOX gene family as marker for multiple-resilience. Pathogens 13, 227. 10.3390/pathogens13030227 PMC10975381 38535570

[erag337-B4] Asai T, Tena G, Plotnikova J, Willmann MR, Chiu WL, Gomez-Gomez L, Boller T, Ausubel FM, Sheen J. 2002. MAP kinase signalling cascade in *Arabidopsis* innate immunity. Nature 415, 977–983. 10.1038/415977a 11875555

[erag337-B5] Balan AS, Tranchina G, Bonanno F, Caruso T, Marra FP, Di Vaio C, Marchese A. 2025. A systems biology framework integrating cross-species transcriptomics and PPI networks for *Xylella fastidiosa* resistance gene identification. BMC Plant Biology 25, 1062. 10.1186/s12870-025-07102-8 PMC12337395 40790398

[erag337-B6] Basile B, Sigillo L, Mataffo A, Corrado G. 2023. Vegetative response to *Verticillium dahliae* of Italian varieties of olive (*Olea europaea* L.). Horticulture, Environment and Biotechnology 64, 943–953.

[erag337-B7] Bazakos C, Alexiou KG, Ramos-Onsins S, et al 2023. Whole genome scanning of a Mediterranean basin hotspot collection provides new insights into olive tree biodiversity and biology. The Plant Journal 116, 303–319. 10.1111/tpj.16270 37164361

[erag337-B8] Ben Sadok I, Celton JM, Essalouh L, El Aabidine AZ, Garcia G, Martinez S, Grati-Kamoun N, Rebai A, Costes E, Khadari B. 2013. QTL mapping of flowering and fruiting traits in olive. PLoS One 8, e62831. 10.1371/journal.pone.0062831 PMC3656886 23690957

[erag337-B9] Boller T, Felix G. 2009. A renaissance of elicitors: perception of microbe-associated molecular patterns and danger signals by pattern-recognition receptors. Annual Review of Plant Biology 60, 379–406. 10.1146/annurev.arplant.57.032905.105346 19400727

[erag337-B10] Boudsocq M, Willmann MR, McCormack M, Lee H, Shan L, He P, Bush J, Cheng SH, Sheen J. 2010. Differential innate immune signalling via Ca^2+^ sensor protein kinases. Nature 464, 418–422. 10.1038/nature08794 PMC2841715 20164835

[erag337-B11] Buonaurio R, Moretti C, da Silva DP, Cortese C, Ramos C, Venturi V. 2015. The olive knot disease as a model to study the role of interspecies bacterial communities in plant disease. Frontiers in Plant Science 6, 434. 10.3389/fpls.2015.00434 PMC4461811 26113855

[erag337-B12] Calle A, Pons-Solé G, Real N, Luque J, Eduardo I, Torguet L, Batlle I, Miarnau X. 2025. Genetic mapping reveals a major locus for red leaf blotch tolerance in almond. Scientia Horticulturae 339, 113901.

[erag337-B13] Campos MD, Zellama MS, Varanda C, Materatski P, Peixe A, Chaouachi M, Félix MDR. 2019. Establishment of a sensitive qPCR methodology for detection of the olive-infecting viruses in Portuguese and Tunisian orchards. Frontiers in Plant Science 10, 694. 10.3389/fpls.2019.00694 PMC6549245 31191591

[erag337-B14] Capriotti AL, Cavaliere C, Foglia P, Piovesana S, Samperi R, Stampachiacchiere S, Lagana A. 2013. Proteomic platform for the identification of proteins in olive (*Olea europaea*) pulp. Analytica Chimica Acta 800, 36–42. 10.1016/j.aca.2013.09.014 24120165

[erag337-B15] Cardoni M, Gómez-Lama Cabanás C, Valverde-Corredor A, Villar R, Mercado-Blanco J. 2022. Unveiling differences in root defense mechanisms between tolerant and susceptible olive cultivars to *Verticillium dahliae*. Frontiers in Plant Science 13, 863055. 10.3389/fpls.2022.863055 PMC9083224 35548299

[erag337-B16] Cardoni M, Mercado-Blanco J. 2023. Confronting stresses affecting olive cultivation from the holobiont perspective. Frontiers in Plant Science 14, 1261754. 10.3389/fpls.2023.1261754 PMC10661416 38023867

[erag337-B17] Cardoni M, Olmo-García L, Serrano-García I, Carrasco-Pancorbo A, Mercado-Blanco J. 2023. The roots of olive cultivars differing in tolerance to *Verticillium dahliae* show quantitative differences in phenolic and triterpenic profiles. Journal of Plant Interactions 18, doi 10.1080/17429145.2023.2206840.

[erag337-B18] Castañeda-Ojeda MP, Moreno-Pérez A, Ramos C, López-Solanilla E. 2017. Suppression of plant immune responses by the *Pseudomonas savastanoi* pv. *savastanoi* NCPPB 3335 type III effector tyrosine phosphatases HopAO1 and HopAO2. Frontiers in Plant Science 8, 680. 10.3389/fpls.2017.00680 PMC5418354 28529516

[erag337-B19] Çetin Ö, Mete N, Ölmez H, Gülen H. 2025. Mapping of QTLs associated with frost tolerance in olive (*Olea europaea* L.). Turkish Journal of Agriculture and Forestry 49, 182–193.

[erag337-B20] Claros MG, Bullones A, Castro AJ, et al 2025. Multi-omic advances in olive tree (*Olea europaea* subsp. *europaea* L.) under salinity: stepping towards ‘smart oliviculture’. Biology 14, 287. 10.3390/biology14030287 PMC11939856 40136543

[erag337-B21] Conti Taguali S, La Spada F, Faraloni C, et al 2025a. Physiological and biochemical responses of olive fruit to *Colletotrichum* spp. infection: a metabolomic insight. Physiological and Molecular Plant Pathology 139, 102844.

[erag337-B22] Conti Taguali S, Riolo M, La Spada F, Cacciola SO, Dionisio G. 2025b. Genome sequencing and assembly of *Phytophthora oleae*, isolate VK10A, causative agent of rot of olive drupes. Journal of Plant Pathology 107, 2345–2350.

[erag337-B23] Corrado G, Alagna F, Rocco M, et al 2012. Molecular interactions between the olive and the fruit fly *Bactrocera oleae*. BMC Plant Biology 12, 86. 10.1186/1471-2229-12-86 PMC3733423 22694925

[erag337-B24] Cruz F, Julca I, Gómez-Garrido J, et al 2016. Genome sequence of the olive tree, *Olea europaea*. GigaScience 5, 29. 10.1186/s13742-016-0134-5 PMC4922053 27346392

[erag337-B25] De Jonge R, van Esse HP, Maruthachalam K, et al 2012. Tomato immune receptor *Ve1* recognizes effector of multiple fungal pathogens uncovered by genome and RNA sequencing. Proceedings of the National Academy of Sciences, USA 109, 5110–5115. 10.1073/pnas.1119623109 PMC3323992 22416119

[erag337-B26] Di Masi S, De Benedetto GE, Malitesta C, Saponari M, Citti C, Cannazza G, Ciccarella G. 2022. HPLC-MS/MS method applied to an untargeted metabolomics approach for the diagnosis of olive quick decline syndrome. Analytical and Bioanalytical Chemistry 414, 465–473. 10.1007/s00216-021-03279-7 PMC8748322 33765220

[erag337-B27] Dong H . 2024. Application of genome editing techniques to regulate gene expression in crops. BMC Plant Biology 24, 100. 10.1186/s12870-024-04786-2 PMC10854132 38331711

[erag337-B28] Estudillo C, Pérez-Rial A, Guerrero-Páez FA, Díez CM, Moral J, Die JV. 2025. Characterization of olive-resistant genes against *Spilocaea oleagina*, the causal agent of scab. Agronomy 15, 452.

[erag337-B29] Fernández-González AJ, Cardoni M, Gómez-Lama Cabanás C, Valverde-Corredor A, Villadas PJ, Fernández-López M, Mercado-Blanco J. 2020. Linking belowground microbial network changes to different tolerance level towards *Verticillium* wilt of olive. Microbiome 8, 11. 10.1186/s40168-020-0787-2 PMC6995654 32007096

[erag337-B30] Fernández-González AJ, Ramírez-Tejero JA, Nevado-Berzosa MP, Luque F, Fernández-López M, Mercado-Blanco J. 2021. Coupling the endophytic microbiome with the host transcriptome in olive roots. Computational and Structural Biotechnology Journal 19, 4777–4789. 10.1016/j.csbj.2021.08.035 PMC8411203 34504670

[erag337-B31] Fernández-González AJ, Serrano A, Luque F, et al 2025. Unveiling essential host genes and keystone microorganisms of the olive tree holobiont linked to *Verticillium* wilt tolerance. Microbiome 13, 239. 10.1186/s40168-025-02216-5 PMC12659499 41299775

[erag337-B32] Gharbi Y, Barkallah M, Bouazizi E, Gdoura R, Triki MA. 2017. Differential biochemical and physiological responses of two olive cultivars differing by their susceptibility to the hemibiotrophic pathogen *Verticillium dahliae*. Physiological and Molecular Plant Pathology 97, 30–39.

[erag337-B33] Gharbi Y, Bouazizi E, Triki MA. 2016. Inductions of defense response in olive plants against *Verticillium dahliae* through application of salicylic acid as abiotic inducer. Journal of Advances in Biology & Biotechnology 5, 1–9.

[erag337-B34] Gharsallah H, Ksentini I, Naayma S, Hadj Taieb K, Abdelhedi N, Schuster C, Triki MA, Ksantini M, Leclerque A. 2020. Identification of fungi in Tunisian olive orchards: characterization and biological control potential. BMC Microbiology 20, 307. 10.1186/s12866-020-01997-z PMC7552492 33046014

[erag337-B35] Giampetruzzi A, Morelli M, Saponari M, Loconsole G, Chiumenti M, Boscia D, Savino VN, Martelli GP, Saldarelli P. 2016. Transcriptome profiling of two olive cultivars in response to infection by the CoDiRO strain of *Xylella fastidiosa* subsp. *pauca*. BMC Genomics 17, 475. 10.1186/s12864-016-2833-9 PMC4924284 27350531

[erag337-B36] Girelli CR, Del Coco L, Angilè F, Scortichini M, Fanizzi FP. 2021. Olive cultivars susceptible or tolerant to *Xylella fastidiosa* subsp. *pauca* exhibit mid-term different metabolomes upon natural infection or a curative treatment. Plants 10, 772. 10.3390/plants10040772 PMC8103516 33920775

[erag337-B37] Glazebrook J . 2005. Contrasting mechanisms of defense against biotrophic and necrotrophic pathogens. Annual Review of Phytopathology 43, 205–227. 10.1146/annurev.phyto.43.040204.135923 16078883

[erag337-B38] Gómez-Lama Cabanás C, Schilirò E, Valverde-Corredor A, Mercado-Blanco J. 2015. Systemic responses in a tolerant olive (*Olea europaea* L.) cultivar upon root colonization by the vascular pathogen *Verticillium dahliae*. Frontiers in Microbiology 6, 928. 10.3389/fmicb.2015.00928 PMC4584997 26441865

[erag337-B39] Granata I, Balan AS, Di Vaio C, et al 2025. First note of QTL mapping of low vigor traits using the updated F2 ‘Koroneiki’ linkage map of olive. Frontiers in Plant Science 16, 1519402. 10.3389/fpls.2025.1519402 PMC11825337 39959349

[erag337-B40] Grasso F, Coppola M, Carbone F, et al 2017. The transcriptional response to the olive fruit fly (*Bactrocera oleae*) reveals extended differences between tolerant and susceptible olive (*Olea europaea* L.) varieties. PLoS One 12, e0183050. 10.1371/journal.pone.0183050 PMC5552259 28797083

[erag337-B41] Jiménez-Ruiz J, Leyva-Pérez MO, Gómez-Lama Cabanás C, Barroso JB, Luque F, Mercado-Blanco J. 2019. The transcriptome of *Verticillium dahliae* responds differentially depending on the disease susceptibility level of the olive (*Olea europaea* L.) cultivar. Genes 10, 251. 10.3390/genes10040251 PMC6523120 30934761

[erag337-B42] Jiménez-Ruiz J, Leyva-Pérez MO, Schilirò E, Barroso JB, Bombarely A, Mueller L, Mercado-Blanco J, Luque F. 2017. Transcriptomic analysis of *Olea europaea* L. roots during the *Verticillium dahliae* early infection process. The Plant Genome 10, plantgenome2016.07.0060. 10.3835/plantgenome2016.07.0060 28464067

[erag337-B43] Jiménez-Ruiz J, Ramírez-Tejero JA, Fernández-Pozo N, et al 2020. Transposon activation is a major driver in the genome evolution of cultivated olive trees (*Olea europaea* L.). The Plant Genome 13, e20010. 10.1002/tpg2.20010 PMC12806974 33016633

[erag337-B44] Jing S, Zhou X, Song Y, Yu D. 2009. Heterologous expression of OsWRKY23 gene enhances pathogen defense and dark-induced leaf senescence in *Arabidopsis*. Plant Growth Regulation 58, 181–190.

[erag337-B45] Jlilat A, Ragone R, Gualano S, Santoro F, Gallo V, Varvaro L, Mastrorilli P, Saponari M, Nigro F, D’Onghia AM. 2021. A non-targeted metabolomics study on *Xylella fastidiosa* infected olive plants grown under controlled conditions. Scientific Reports 11, 1070. 10.1038/s41598-020-80090-x PMC7806896 33441842

[erag337-B46] Jones JDG, Dangl JL. 2006. The plant immune system. Nature 444, 323–329. 10.1038/nature05286 17108957

[erag337-B47] Julca I, Marcet-Houben M, Cruz F, Gómez-Garrido J, Gaut BS, Díez CM, Gut IG, Alioto TS, Vargas P, Gabaldón T. 2020. Genomic evidence for recurrent genetic admixture during the domestication of Mediterranean olive trees (*Olea europaea* L.). BMC Biology 18, 148. 10.1186/s12915-020-00881-6 PMC7586694 33100219

[erag337-B48] Kaya AC, İpek M, İpek A, Gündoğdu MA, Aktepe Tangu N, Teoman Duran S, Şeker M, Akbulut M. 2024. QTL mapping for fatty acid composition in olive oil using a high-density genetic map based on SNP markers. Turkish Journal of Agriculture and Forestry 48, 490–501.

[erag337-B49] Keykhasaber M, Thomma BPHJ, Hiemstra JA. 2018. Verticillium wilt caused by *Verticillium dahliae* in woody plants with emphasis on olive and shade trees. European Journal of Plant Pathology 150, 21–37.

[erag337-B50] Kourelis J, van der Hoorn RAL. 2018. Defended to the nines: 25 years of resistance gene cloning identifies nine mechanisms for R protein function. The Plant Cell 30, 285–299. 10.1105/tpc.17.00579 PMC5868693 29382771

[erag337-B51] Lagiotis G, Karamichali I, Astrinaki M, et al 2024. High-resolution gene expression atlases of two contrasting major Greek olive (*Olea europaea* L.) tree cultivars for oil and table olive production. Physiologia Plantarum 176, e14600. 10.1111/ppl.14600 PMC11659808 39501529

[erag337-B52] Lamoumni O, El Bakkali A, Abou Saaid O, et al 2025. QTL mapping and candidate gene identification of the full flowering date trait in olive trees (*Olea europaea* L.). BMC Plant Biology 25, 1768. 10.1186/s12870-025-07171-9 PMC12750909 41272424

[erag337-B53] La Notte P, Saponari M, Mousavi S, et al 2024. A survey in natural olive resources exposed to high inoculum pressure indicates the presence of traits of resistance to *Xylella fastidiosa* in Leccino offspring. Frontiers in Plant Science 15, 1457831. 10.3389/fpls.2024.1457831 PMC11471571 39403622

[erag337-B54] Lantero E, Matallanas B, Callejas C. 2023. Current status of the main olive pests: useful integrated pest management strategies and genetic tools. Applied Sciences 13, 12078.

[erag337-B55] Le Roux PM, Khan MA, Broggini GA, Duffy B, Gessler C, Patocchi A. 2010. Mapping of quantitative trait loci for fire blight resistance in the apple cultivars ‘Florina’ and ‘Nova easygro’. Genome 53, 710–722. 10.1139/g10-047 20924420

[erag337-B56] Leyva-Pérez MO, Jiménez-Ruiz J, Gómez-Lama Cabanás C, Valverde-Corredor A, Barroso JB, Luque F, Mercado-Blanco J. 2018. Tolerance of olive (*Olea europaea*) cv Frantoio to *Verticillium dahliae* relies on both basal and pathogen-induced differential transcriptomic responses. New Phytologist 217, 671–686. 10.1111/nph.14833 29023762

[erag337-B57] Li N, Han X, Feng D, Yuan D, Huang LJ. 2019. Signaling crosstalk between salicylic acid and ethylene/jasmonate in plant defense: do we understand what they are whispering? International Journal of Molecular Sciences 20, 671. 10.3390/ijms20030671 PMC6387439 30720746

[erag337-B58] Li N-Y, Zhou L, Zhang D-D, et al 2018. Heterologous expression of the cotton NBS-LRR gene GbaNA1 enhances Verticillium wilt resistance in *Arabidopsis*. Frontiers in Plant Science 9, 119. 10.3389/fpls.2018.00119 PMC5808209 29467784

[erag337-B59] Licciardello G, Mosca A, Di Silvestro S, Puglisi D, Russo MP, Catara V, Caruso P. 2023. Cultivar susceptibility to olive knot disease and association with endophytic microbiota community. Agronomy 13, 468.

[erag337-B60] López-Moral A, Llorens E, Scalschi L, García-Agustín P, Trapero A, Agustí-Brisach C. 2022. Resistance induction in olive tree (*Olea europaea*) against Verticillium wilt by two beneficial microorganisms and a copper phosphite fertilizer. Frontiers in Plant Science 13, 831794. 10.3389/fpls.2022.831794 PMC8905222 35283881

[erag337-B61] Luvisi A, Aprile A, Sabella E, Vergine M, Nicolì F, Nutricati E, Miceli A, Negro C, De Bellis L. 2017a. *Xylella fastidiosa* subsp. *pauca* (CoDiRO strain) infection in four olive (*Olea europaea* L.) cultivars: profile of phenolic compounds in leaves and progression of leaf scorch symptoms. Phytopathologia Mediterranea 56, 259–273.

[erag337-B62] Luvisi A, Nicolì F, De Bellis L. 2017b. Sustainable management of plant quarantine pests: the case of olive quick decline syndrome. Sustainability 9, 659.

[erag337-B63] Lv J, Jiang C, Wu W, et al 2024. The gapless genome assembly and multi-omics analyses unveil a pivotal regulatory mechanism of oil biosynthesis in the olive tree. Horticulture Research 11, uhae168. 10.1093/hr/uhae168 PMC11300844 39108575

[erag337-B64] Marchese A, Balan B, Trippa DA, Bonanno F, Caruso T, Imperiale V, Marra FP, Giovino A. 2023b. NGS transcriptomic analysis uncovers the possible resistance mechanisms of olive to *Spilocaea oleagina* leaf spot infection. Frontiers in Plant Science 14, 1219580. 10.3389/fpls.2023.1219580 PMC10388255 37528972

[erag337-B65] Marchese A, Bonanno F, Marra FP, et al 2023a. Recovery and genotyping ancient Sicilian monumental olive trees. Frontiers in Conservation Science 4, 1206832.

[erag337-B66] Martí JM, Arias-Giraldo LF, Díaz-Villanueva W, Arnau V, Rodríguez-Franco A, Garay CP. 2020. Metatranscriptomic dynamics after *Verticillium dahliae* infection and root damage in *Olea europaea*. BMC Plant Biology 20, 79. 10.1186/s12870-019-2185-0 PMC7027230 32066386

[erag337-B67] Martinelli F, Marchese A, Giovino A, Marra FP, Della Noce I, Caruso T, Dandekar AM. 2019. In-field and early detection of *Xylella fastidiosa* infections in olive using a portable instrument. Frontiers in Plant Science 9, 2007. 10.3389/fpls.2018.02007 PMC6345699 30713547

[erag337-B68] Mascagni F, Barghini E, Ceccarelli M, Baldoni L, Trapero C, Díez CM, Natali L, Cavallini A, Giordani T. 2022. The singular evolution of *Olea* genome structure. Frontiers in Plant Science 13, 869048. 10.3389/fpls.2022.869048 PMC9009077 35432417

[erag337-B69] Mascuñano B, Coto-Elena J, Guerrero-Sánchez VM, et al 2025. Transcriptome analysis of wild olive (*Olea europaea* L. subsp. *europaea* var. *sylvestris*) clone AC18 provides insight into the role of lignin as a constitutive defense mechanism underlying resistance to Verticillium wilt. BMC Plant Biology 25, 292. 10.1186/s12870-025-06301-7 PMC11884133 40045216

[erag337-B70] Montes-Osuna N, Mercado-Blanco J. 2020. Verticillium wilt of olive and its control: what did we learn during the last decade? Plants 9, 735. 10.3390/plants9060735 PMC7356185 32545292

[erag337-B71] Morelli M, García-Madero JM, Jos Á, et al 2021. *Xylella fastidiosa* in olive: a review of control attempts and current management. Microorganisms 9, 1771. 10.3390/microorganisms9081771 PMC8397937 34442850

[erag337-B72] Moretti C, Rezzonico F, Orfei B, et al 2021. Synergistic interaction between the type III secretion system of the endophytic bacterium *Pantoea agglomerans* DAPP-PG 734 and the virulence of the causal agent of olive knot *Pseudomonas savastanoi* pv. *savastanoi* DAPP-PG 722. Molecular Plant Pathology 22, 1209–1225. 10.1111/mpp.13105 PMC8435235 34268839

[erag337-B73] Movahedi A, Aghaei-Dargiri S, Barati B, Kadkhodaei S, Wei H, Sangari S, Yang L, Xu C. 2022. Plant immunity is regulated by biological, genetic, and epigenetic factors. Agronomy 12, 2790.

[erag337-B74] Novelli S, Gismondi A, Di Marco G, Canuti L, Nanni V, Canini A. 2019. Plant defense factors involved in *Olea europaea* resistance against *Xylella fastidiosa* infection. Journal of Plant Research 132, 439–455. 10.1007/s10265-019-01108-8 30993555

[erag337-B75] Pagán I, García-Arenal F. 2018. Tolerance to plant pathogens: theory and experimental evidence. International Journal of Molecular Sciences 19, 810. 10.3390/ijms19030810 PMC5877671 29534493

[erag337-B76] Palomo-Ríos E, Narváez I, Pliego-Alfaro F, Mercado JA. 2021. Olive (*Olea europaea* L.) genetic transformation: current status and future prospects. Genes 12, 386. 10.3390/genes12030386 PMC7998262 33803172

[erag337-B77] Parvez S, Asif M, Ahmad A, et al 2025. Tracing the path from conservation to expansion: evolutionary insights into NLR genes in Oleaceae. BMC Plant Biology 25, 259. 10.1186/s12870-025-06233-2 PMC11863892 40000960

[erag337-B78] Pieterse CMJ, Van der Does D, Zamioudis C, Leon-Reyes A, Van Wees SCM. 2012. Hormonal modulation of plant immunity. Annual Review of Cell and Developmental Biology 28, 489–521. 10.1146/annurev-cellbio-092910-154055 22559264

[erag337-B79] Poveda J, Baptista P. 2021. Filamentous fungi as biocontrol agents in olive (*Olea europaea* L.) diseases: mycorrhizal and endophytic fungi. Crop Protection 146, 105672.

[erag337-B80] Rai MF, Tycksen ED, Sandell LJ, Brophy RH. 2018. Advantages of RNA-Seq compared to RNA microarrays for transcriptome profiling of anterior cruciate ligament tears. Journal of Orthopaedic Research 36, 484–497. 10.1002/jor.23661 PMC5787041 28749036

[erag337-B81] Randazzo CL, Todaro A, Pino A, Pitino I, Corona O, Caggia C. 2017. Microbiota and metabolome during controlled and spontaneous fermentation of *Nocellara etnea* table olives. Food Microbiology 65, 136–148. 10.1016/j.fm.2017.01.022 28399996

[erag337-B82] Rao G, Zhang J, Liu X, Lin C, Xin H, Xue L, Wang C. 2021. De novo assembly of a new *Olea europaea* genome accession using nanopore sequencing. Horticulture Research 8, 64. 10.1038/s41438-021-00498-y PMC8012569 33790235

[erag337-B83] Rapicavoli J, Ingel B, Blanco-Ulate B, Cantu D, Roper C. 2018a. *Xylella fastidiosa*: an examination of a re-emerging plant pathogen. Molecular Plant Pathology 19, 786–800. 10.1111/mpp.12585 PMC6637975 28742234

[erag337-B84] Rapicavoli JN, Blanco Ulate B, Muszyński A, et al 2018b. Lipopolysaccharide O antigen delays plant innate immune recognition of *Xylella fastidiosa*. Nature Communications 9, 390. 10.1038/s41467-018-02861-5 PMC5786101 29374171

[erag337-B85] Ren C, Mohamed MSM, Aini N, Kuang Y, Liang Z. 2024. CRISPR/Cas in grapevine genome editing: the best is yet to come. Horticulturae 10, 965.

[erag337-B86] Riolo M, Luz C, Santilli E, Meca G, Cacciola SO. 2023. Secondary metabolites produced by four *Colletotrichum* species in vitro and on fruits of diverse olive cultivars. Fungal Biology 127, 1118–1128. 10.1016/j.funbio.2023.06.003 37495302

[erag337-B87] Sabella E, Luvisi A, Aprile A, Negro C, Vergine M, Nicolì F, Miceli A, De Bellis L. 2018. *Xylella fastidiosa* induces differential expression of lignification related-genes and lignin accumulation in tolerant olive trees cv. Leccino. Journal of Plant Physiology 220, 60–68. 10.1016/j.jplph.2017.10.007 29149645

[erag337-B88] Saponari M, Boscia D, Nigro F, Martelli GP. 2013. Identification of DNA sequences related to *Xylella fastidiosa* in oleander, almond and olive trees exhibiting leaf scorch symptoms in Apulia (Southern Italy). Journal of Plant Pathology 95, 659–668.

[erag337-B89] Saponari M, Giampetruzzi A, Loconsole G, Boscia D, Saldarelli P. 2019. *Xylella fastidiosa* in olive in Apulia: where we stand. Phytopathology 109, 175–186. 10.1094/PHYTO-08-18-0319-FI 30376439

[erag337-B90] Sebastiani L, Sarfraz I, Francini A, Celii M, Wing RA, Zuccolo A. 2025. PacBio genome assembly of *Olea europaea* L. subsp. *europaea* cultivars Frantoio and Leccino. Scientific Data 12, 1095. 10.1038/s41597-025-05363-4 PMC12217686 40593836

[erag337-B91] Sekhon RS, Briskine R, Hirsch CN, Myers CL, Springer NM, Buell CR, de Leon N, Kaeppler SM. 2013. Maize gene atlas developed by RNA sequencing and comparative evaluation of transcriptomes based on RNA sequencing and microarrays. PLoS One 8, e61005. 10.1371/journal.pone.0061005 PMC3634062 23637782

[erag337-B92] Serrano A, León L, Belaj A, Román B. 2020. Nucleotide diversity analysis of candidate genes for Verticillium wilt resistance in olive. Scientia Horticulturae 274, 109653.

[erag337-B93] Talhinhas P, Loureiro A, Oliveira H. 2018. Olive anthracnose: a yield- and oil quality-degrading disease caused by several species of *Colletotrichum* that differ in virulence, host preference and geographical distribution. Molecular Plant Pathology 19, 1797–1807. 10.1111/mpp.12676 PMC6638118 29517840

[erag337-B94] Torres MA, Jones JDG, Dangl JL. 2006. Reactive oxygen species signaling in response to pathogens. Plant Physiology 141, 373–378. 10.1104/pp.106.079467 PMC1475467 16760490

[erag337-B95] Uceda-Campos G, Feitosa-Junior OR, Santiago CRN, et al 2022. Comparative genomics of *Xylella fastidiosa* explores candidate host-specificity determinants and expands the known repertoire of mobile genetic elements and immunity systems. Microorganisms 10, 914. 10.3390/microorganisms10050914 PMC9148166 35630358

[erag337-B96] Unver T, Wu Z, Sterck L, et al 2017. Genome of wild olive and the evolution of oil biosynthesis. Proceedings of the National Academy of Sciences, USA 114, E9413–E9422. 10.1073/pnas.1708621114 PMC5676908 29078332

[erag337-B97] Vergine M, Vita F, Casati P, Passera A, Ricciardi L, Pavan S, Aprile A, Sabella E, De Bellis L, Luvisi A. 2024. Characterization of the olive endophytic community in genotypes displaying a contrasting response to *Xylella fastidiosa*. BMC Plant Biology 24, 337. 10.1186/s12870-024-04980-2 PMC11044560 38664617

[erag337-B98] Wang C, Ma Z, Zhou J, et al 2024. A simple and effective VIGS system facilitates the control of citrus canker by silencing *CsLOB1*. Phytopathology Research 6, 12.

[erag337-B99] Wang S, Zhao S, Du M, Mo K, Pang S, Zou X. 2024. Development and application of gene editing in citrus: how and what to do. Horticulture Advances 2, 31.

[erag337-B100] Wang T, Cao H, Du T, Meng D, Yang Q, Li J, Zhang J, Zeng Y. 2024. Integrated transcriptome and metabolome analysis revealed the key role of the flavonoid biosynthesis in olive defense against *Alternaria alternata*. Physiologia Plantarum 176, e14529. 10.1111/ppl.14529 39319936

[erag337-B101] Wöhner TW, Flachowsky H, Richter K, Garcia Libreros T, Trognitz F, Hanke MV, Peil A. 2014. QTL mapping of fire blight resistance in *Malus × robusta* 5 after inoculation with different strains of *Erwinia amylovora*. Molecular Breeding 34, 217–230.

[erag337-B102] Young ND . 1996. QTL mapping and quantitative disease resistance in plants. Annual Review of Phytopathology 34, 479–501. 10.1146/annurev.phyto.34.1.479 15012553

[erag337-B103] Zipfel C, Robatzek S, Navarro L, Oakeley EJ, Jones JDG, Felix G, Boller T. 2004. Bacterial disease resistance in *Arabidopsis* through flagellin perception. Nature 428, 764–767. 10.1038/nature02485 15085136

